# A Late Role for *bmp2b* in the Morphogenesis of Semicircular Canal Ducts in the Zebrafish Inner Ear

**DOI:** 10.1371/journal.pone.0004368

**Published:** 2009-02-03

**Authors:** Katherine L. Hammond, Helen E. Loynes, Catriona Mowbray, Greg Runke, Matthias Hammerschmidt, Mary C. Mullins, Victoria Hildreth, Bill Chaudhry, Tanya T. Whitfield

**Affiliations:** 1 MRC Centre for Developmental and Biomedical Genetics and Department of Biomedical Science, University of Sheffield, Sheffield, United Kingdom; 2 Max-Planck-Institute of Immunobiology, Freiburg, Germany; 3 Department of Cell and Developmental Biology, University of Pennsylvania School of Medicine, Philadelphia, Pennsylvania, United States of America; 4 Institute of Human Genetics, Newcastle University, Newcastle upon Tyne, United Kingdom; Harvard University, United States of America

## Abstract

**Background:**

The Bone Morphogenetic Protein (BMP) genes *bmp2* and *bmp4* are expressed in highly conserved patterns in the developing vertebrate inner ear. It has, however, proved difficult to elucidate the function of BMPs during ear development as mutations in these genes cause early embryonic lethality. Previous studies using conditional approaches in mouse and chicken have shown that Bmp4 has a role in semicircular canal and crista development, but there is currently no direct evidence for the role of Bmp2 in the developing inner ear.

**Methodology/Principal Findings:**

We have used an RNA rescue strategy to test the role of *bmp2b* in the zebrafish inner ear directly. Injection of *bmp2b* or *smad5* mRNA into homozygous mutant *swirl* (*bmp2b^−/−^*) embryos rescues the early patterning defects in these mutants and the fish survive to adulthood. As injected RNA will only last, at most, for the first few days of embryogenesis, all later development occurs in the absence of *bmp2b* function. Although rescued *swirl* adult fish are viable, they have balance defects suggestive of vestibular dysfunction. Analysis of the inner ears of these fish reveals a total absence of semicircular canal ducts, structures involved in the detection of angular motion. All other regions of the ear, including the ampullae and cristae, are present and appear normal. Early stages of otic development in rescued *swirl* embryos are also normal.

**Conclusions/Significance:**

Our findings demonstrate a critical late role for *bmp2b* in the morphogenesis of semicircular canals in the zebrafish inner ear. This is the first demonstration of a developmental role for any gene during post-embryonic stages of otic morphogenesis in the zebrafish. Despite differences in the early stages of semicircular canal formation between zebrafish and amniotes, the role of Bmp2 in semicircular canal duct outgrowth is likely to be conserved between different vertebrate species.

## Introduction

The inner ears of all vertebrates detect both auditory and vestibular stimuli to a lesser or greater extent, depending on the species. The region of the ear responsible for detection of rotational motion (angular acceleration) consists of three orthogonally arranged semicircular canals and their associated sensory patches, the cristae. Angular motion stimuli normally cause displacement of sensory hair cells in the cristae due to the inertia of the fluid in the semicircular canals, and compensatory muscle movements (the vestibular righting reflex) allow an animal to maintain postural equilibrium as it turns. The semicircular canal system is well conserved among jawed vertebrates; in the embryo, the developing canal ducts and cristae have been shown to express members of the Bone Morphogenetic Protein (BMP) family of signalling molecule genes in all vertebrate species examined. *bmp4* is expressed in the developing cristae of zebrafish, mouse and chick [1]–[4], while in mouse and chick, *Bmp2* is expressed in the canal genesis zone, adjacent to the cristae, and in the epithelium of the developing canals [5]. Similarly, in zebrafish, *bmp2b* is expressed in the developing cristae, and by 72 hours post fertilisation (hpf) expression is also detected in the epithelial projections forming the semicircular canals [1], [6]. *Bmp7* is expressed strongly in epithelium of the developing semicircular canal system in both chick [7] and zebrafish (*bmp7b*) [8].

Despite the conserved expression of *Bmp* genes in the developing ear, it has been difficult to assess BMP function during inner ear development using a conventional genetic approach, as BMP signalling plays a vital role in establishing ventral identity in the early embryo. Thus mutations in genes encoding BMP pathway components often cause early embryonic lethality [9]–[11]. To circumvent this early requirement for BMP signalling in the embryo, it is therefore necessary to use a conditional approach to restrict BMP disruption either to later developmental stages or to tissues of the developing ear. Ectopic application of the BMP antagonists Noggin or DAN to the developing chick inner ear showed that BMPs have a role in the formation of cristae and semicircular canal ducts: both structures are variably absent in treated embryos [12]–[14]. These experiments did not, however, indicate which BMPs are involved. More recently, Wu and colleagues [15] have used Cre-*lox* technology and electroporation techniques to knock down *Bmp4* function specifically in the inner ears and cristae of mouse and chick embryos, respectively. This work clearly demonstrates an important role for *Bmp4* in semicircular canal and crista development, with the most severely affected embryos having no canal ducts or cristae together with malformed saccules and utricles [15].

The study by Chang et al. suggests that the effect of Bmp4 on canal development in the mouse and chick is mediated by *Bmp2*, as *Bmp2* expression is down-regulated when *Bmp4* is knocked down [15]. This follows from previous work by the same group implicating *Bmp2* in canal development [5], [7]. In the chick, a canal genesis zone adjacent to the crista expresses *Bmp2* under the control of FGF signalling. Ectopic FGF treatments resulted in ectopic *Bmp2* expression and a failure of resorption at the canal fusion plate (and thus excess canal duct tissue), while reduced FGF signalling resulted in reduced *Bmp2* expression and a lack of semicircular canal ducts, although the crus commune and some ampullae were still formed [5]. Crucially, application of Noggin alongside FGF rescued the canal duct phenotype, suggesting that the promotion of canal duct outgrowth by FGF is mediated through *Bmp2*. While these data strongly suggest that *Bmp2* is required for semicircular canal development, they do not, however, provide direct evidence.

We have used an alternative approach in the zebrafish to test the role of *bmp2b* directly, exploiting the ability to rescue the early defects in BMP pathway mutants by mRNA injection. Homozygous zebrafish *swirl* (*swr/bmp2b*) mutant embryos are severely dorsalised and lyse at the 13–14 somite stage (around 16 hpf) due to pressure on the yolk [9], [11], [16]. This corresponds to the otic placode stage in the developing embryo, but as a result of the dorsalisation, otic placodal tissue is severely reduced or absent from these embryos [11]. However, injection of in vitro-synthesised *bmp2b* or *smad5* mRNA at the 1–2 cell stage results in complete rescue of the dorsoventral patterning defects, allowing the homozygous mutant fish to reach adulthood [9], [11]. As the injected mRNA is only expected to last for the first few days of embryogenesis, all subsequent developmental stages are completed in the absence of *bmp2b* function. As we show here, rescued homozygous *swr/bmp2b^−/−^* adults have severe structural abnormalities of the inner ear and concomitant balance defects, revealing a critical late requirement for *bmp2b* during semicircular canal morphogenesis in the zebrafish.

## Results

### Rescued homozygous *swr/bmp2b* adult fish have an abnormal swimming behaviour indicative of vestibular dysfunction

Rescued homozygous *swr/bmp2b* mutant fish are adult viable and fertile, and appear grossly morphologically normal, but display a very specific and fully penetrant abnormal swimming behaviour (data not shown). They swim normally except when making sudden changes in direction: instead of making a neat turn they lose their dorsoventral orientation momentarily and perform a messy somersault. This is particularly evident when the fish are excited or disturbed, for example when they are about to be fed. This indicates a failure or delay in the detection of angular motion stimuli, or in the vestibular righting reflex that maintains normal postural control as the fish turns. The abnormal swimming behaviour is based on independent observations of several batches of rescued *swr/bmp2b* mutant fish (>100 individuals); 100% of rescued adults swam abnormally. Such behaviour is similar to, but not as severe as, that of fish that cannot balance correctly due to defects in the sensory hair cells of the inner ear hair [17]. It is worth noting, however, that *bmp2b*-rescued *swr^ta72^* fish (n = 5) show a wild-type dorsal light reflex, indicating that they retain the ability to sense, and orient themselves with respect to, gravity (see Materials and Methods; data not shown).

### The inner ears of adult rescued *swr/bmp2b* fish lack semicircular canal ducts, but all sensory epithelia are present

**Figure 1:**
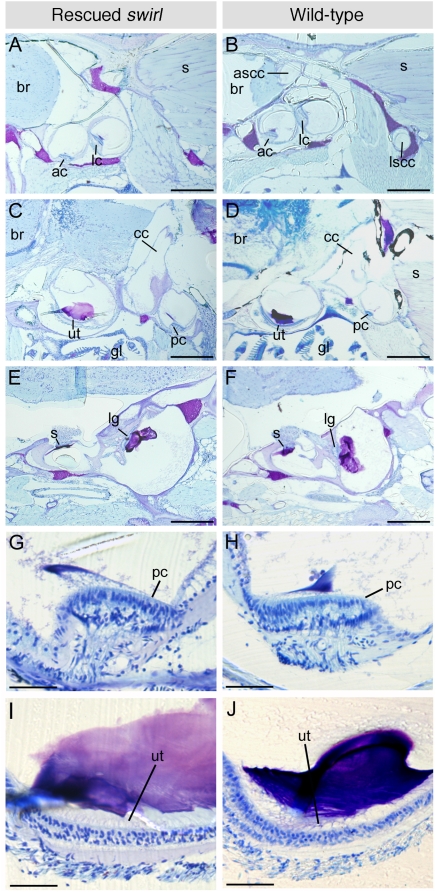
Semicircular canal ducts are absent, but sensory patches are present, in the inner ears of adult rescued *swr* zebrafish. (A–F) 10 µm resin parasagittal sections through the inner ears of adult rescued *swr* zebrafish and age-matched wild-types (anterior to the left, dorsal to the top). Note the absence of semicircular canal ducts in the rescued *swr* ear (A), which are clearly present in the wild-type ear in an equivalent section (B, showing lumens of anterior and posterior canal ducts). All sensory patches detected in the wild-type ears are also present in the rescued *swr* ears. Differences in the orientation of the lagenar macula (E, F) reflect a slight difference in the position of the wild-type and *swr* sections for this pair of panels. (G–J) Higher magnification views of the posterior crista (G, H) and utricular macula (I, J). The structure of the sensory patches is similar in both rescued *swr* and wild-type ears. Abbreviations: ac, anterior crista; lc, lateral crista; pc, posterior crista; ascc, anterior semicircular canal duct; lscc, lateral semicircular canal duct; cc, crus commune; ut, utricular macula; s, saccular macula; lg, lagenar macula; br, brain; s, somite; gl, gill. Scale bar, (A–F) 400 µm; (G–J) 50 µm.

To investigate the causes of the behavioural defect further, we analysed inner ear morphology in the rescued *swr* adults by histological sectioning. We cut resin sections at 10 µm through the heads of six rescued homozygous adult *swr* fish (three *bmp2b*-rescued *swr^ta72^*, two *smad5*-rescued *swr^tdc24^* and one *smad5*-rescued *swr^tc300^*) and three age-matched wild-type fish. Examination of these sections indicated that the semicircular canal ducts were absent in all six rescued *swr* mutant specimens (Fig. 1). The ampullae, utricule, saccule and lagena were all present, however, and contained sensory patches (cristae, utricular macula, saccular macula and lagenar macula, respectively) of apparently normal morphology (Fig. 1).

**Figure 2:**
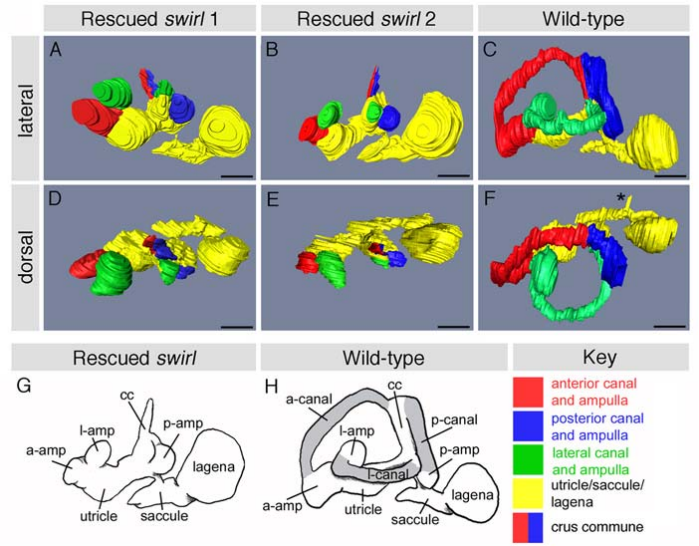
Three-dimensional reconstructions of adult rescued *swr* inner ears. (A–F) Three-dimensional reconstructions of the inner ears of two adult rescued *swr* fish (*swirl* 1 (A, D) and *swirl* 2 (B, E)) and an age-matched wild-type fish (C, F). Semicircular canals are absent in the rescued *swr* fish ears, while the ampullae, crus commune, utricle, saccule and lagena are present and appear normal. Note that the pars superior (ampullae, crus commune and utricle) of *swirl* 1 is twisted relative to the pars inferior (saccule and lagena), about the point marked with the arrow (we are unable to tell whether or not this is an experimental artefact). The kink in the lateral canal of the wild-type ear (C) is an artefact. The asterisk marks the transverse canal that links the two saccules across the midline. (G, H) Sketch of the inner ears of adult rescued *swr* and wild-type fish. Shading in the wild-type ear indicates the regions missing from rescued *swr* ears. Abbrevations: a-amp, anterior ampulla; l-amp, lateral ampulla; p-amp, posterior ampulla; a-canal, anterior semicircular canal; l-canal, lateral semicircular canal; p-canal, posterior semicircular canal; cc, crus commune. *swirl 1* is a *smad5*-rescued *swr^dc24^* fish; *swirl 2* is a *bmp2b*-rescued *swr^ta72^* fish. A–C, G and H are lateral views of left hand ears, with anterior to the left; D–F are dorsal views. Scale bar, 500 µm.

To obtain a clearer picture of the morphological defects, we used the sections to generate three-dimensional reconstructions of the inner ears of three rescued *swr* fish (two *bmp2b*-rescued *swr^ta72^* and one *smad5*-rescued *swr^tdc24^*) and two wild-type fish. We obtained full reconstructions of three rescued *swr* ears from two fish and two partial reconstructions from a single fish. We also obtained three fully reconstructed ears from the two wild-type fish (Fig. 2 and Supplementary Videos S1, S2). The reconstructions confirmed that all three rescued *swr* specimens (five ears examined) lacked semicircular canal ducts. The ampullae, crus commune and the posterior end of the lateral canal were present in all cases, but ended blindly. In the three rescued *swr* ears in which the vestibular chambers were reconstructed, the utricule, saccule and lagena were all present and grossly normal in size and morphology (Fig. 2 and Supplementary Videos S1, S2).

### Sensory patch and semicircular canal development are normal in the inner ears of rescued *swr/bmp2b* fish during embryonic stages

**Figure 3:**
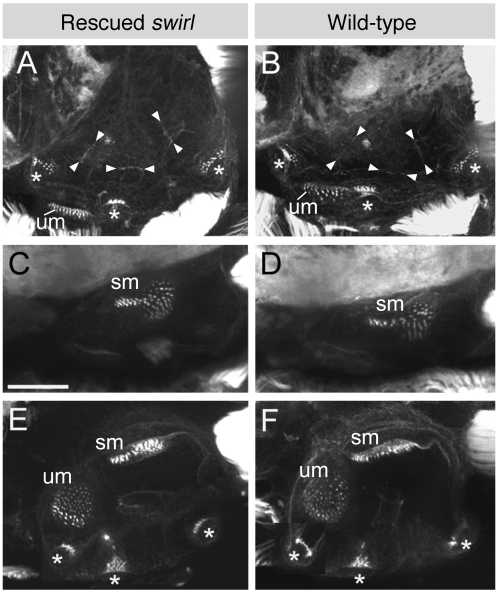
Sensory patches and fusion plates are present in the inner ears of 5 dpf rescued *swr* embryos. Projected confocal z-stacks through inner ears of 5 dpf embryos stained with FITC-conjugated phalloidin, revealing the actin-rich stereociliary bundles of the sensory hair cells and the cortical actin in every cell. (A–D) Lateral views; anterior to left, dorsal to top. (A, B) Lateral plane of focus, showing the cristae, utricular macula and the fusion plates (arrowheads) between the semicircular canal projections. (C, D) More medial plane of focus showing the saccular macula. (E, F) Dorsal views; anterior to left, medial to top. Images shown are composites of two sets of projected z-stacks as the anterior crista is in a more dorsal plane of focus than the remaining sensory patches. Abbreviations: sm, saccular macula; um, utricular macula. Cristae are indicated with an asterisk. Scale bar, 50 µm.

The absence of canal duct tissue in adult fish led us to examine otic development in rescued *swr* mutants at embryonic and early larval stages. To analyse the development of sensory patches and semicircular canals, we stained *smad5*-RNA rescued *swr^ta72^* and *swr^tdc24^* embryos and wild-type controls at 48 hpf to 7 dpf (days post fertilisation) with FITC-conjugated phalloidin. This marks both cortical actin and the actin-rich stereocilia of sensory hair cell bundles, allowing both general ear morphology and the sensory patches to be visualised (see, for example, [18]). In the wild-type ear at 48 hpf, hair cells have differentiated in two sensory patches, the utricular and saccular maculae. At the same stage, projections of epithelium that will form the hubs of the semicircular canals begin to ingress into the vesicle, meeting at a fusion plate to form pillars by approximately 72 hpf. Hair cells in the cristae are present by 60 hpf [19]. At all stages examined, both sensory patch morphology and semicircular canal development appeared normal in the rescued *swr* ears, including the formation of semicircular canal fusion plates (Fig. 3), although we cannot rule out the presence of any subtle defects. Of thirteen rescued *swr* embryos examined (four at 48 hpf, two at 72 hpf, five at 5 dpf and two at 7 dpf) only one of the 5 dpf fish did not show a wild-type phenotype. In the ears of this fish, all sensory patches and semicircular canal pillars were present, but the posterior macula was disorganised (data not shown). As the balance defect in rescued *swr* adults is fully penetrant, and all six sets of adult sections examined show a consistent phenotype, the posterior macula defect in this individual is likely to be non-specific rather than due to a specific lack of *bmp2b*. Overall, our data demonstrate a late requirement for *bmp2b* in the development of semicircular canals in the zebrafish that becomes manifest after 7 dpf.

## Discussion

### Morphological defects in the ears of rescued *swr/bmp2b* mutant fish are consistent with their behavioural abnormalities

Several adult viable zebrafish lines have previously been described that exhibit abnormal swimming behaviour similar to, but more severe than, that of the rescued *swr* fish described here. These ‘circler’ mutants have defects in the fine structure of the sensory hair cells, and identification of the causative mutations in these lines has revealed genes with roles in hair cell structure and function [17], [20]–[26]. All hair cells (of the maculae and cristae in the ear, and neuromasts in the lateral line system) are affected in the circler mutant lines, and homozygous viable adult fish exhibit a potentiated dorsal light reflex, indicating a loss of gravistatic postural control [17]. In contrast, in the adult rescued *swr* fish, we see a gross structural abnormality in the inner ear that is robust, highly specific and consistent with the observed behavioural defects. Although ampullae and cristae are present in the ears of rescued *swr* adults, the associated non-sensory epithelium forming the semicircular canal ducts is absent. The cristae would therefore be unable to receive correct angular motion stimuli, accounting for the failure of the vestibular righting reflex and loss of postural control whilst turning. The sensory patches responsible for detection of gravitoinertial stimuli—the lagenar, saccular and utricular maculae—are all present in grossly normal vestibular chambers, accounting for the normal dorsal light reflex and gravistatic postural control.

### *bmp2b* has a late, highly specific role in morphogenesis of the semicircular canal ducts of the zebrafish inner ear

The requirement for *bmp2b* function for correct morphogenesis of the semicircular ducts is the first demonstration of a developmental role for any gene during post-embryonic stages of otic morphogenesis in the zebrafish. We see a very consistent phenotype in all six fish examined, in contrast to the variable phenotypes obtained by conditional approaches in other species. Previous studies in other species implicate BMPs, in particular Bmp4, in both crista and semicircular canal development during embryogenesis: both structures are affected when *Bmp4* is knocked down specifically in mouse and chicken inner ears [15]. However, it has been suggested that the effect of *Bmp4*, *Fgf3* and *Fgf10* (all expressed in the cristae) on semicircular canal development may be mediated by *Bmp2*, which is expressed in a canal genesis zone adjacent to the crista in mouse and chicken [5], [15]. Thus non-sensory development may require the prior specification of sensory tissue. Our data, which demonstrate that zebrafish *bmp2b* has a highly specific late role in semicircular canal morphogenesis, are consistent with this model.

Several other factors, including Wnt signalling from the dorsal hindbrain and *Hmx*, *Dlx*, *Prx* and *Netrin1* genes in the ear, are also implicated in vestibular morphogenesis in mouse and chick [27]–[32]. Mutations in several of these genes, however, have widespread effects on both sensory and non-sensory epithelia in the ear. It is worth noting that some of the effects of these genes may be mediated by BMP signalling; down-regulation of *Wnt*, *Hmx* or *Dlx* genes results in down-regulation or disorganisation of *Bmp4* expression in the developing cristae [27]–[29], [33]. Equally, however, some are affected by manipulation of Bmp4 levels; in particular, *Dlx5* is down-regulated when Bmp4 function is absent from the ear [15].

### BMPs are likely to signal through Alk8 in the zebrafish ear

Several other homozygous mutants of the BMP signalling pathway have been rescued to adulthood by mRNA injection, including *snh/bmp7a* [34], [35], *laf/alk8* [36] and *sbn/smad5* [37], [38], but few other late (post-embryonic) phenotypes affecting organ systems have been revealed by mRNA rescue. One example is provided by the rescued *laf/alk8* adult fish, which—unlike rescued *swr/bmp2b* adults—have stunted growth and an enlarged heart [36]. Interestingly, these fish also display a very similar abnormal swimming behaviour to that described here for the rescued *swr/bmp2b* adults [36] (K. Mintzer and MCM, unpublished observation), suggesting that they may have similar inner ear defects. Rescued homozygous *smad5* and *bmp7a* mutant adults, however, appear to swim and balance normally (MCM and MH, unpublished observations).

*alk8* codes for a type 1 BMP receptor, through which Bmp2b and other BMPs act; the similar behavioural phenotype in rescued *swr/bmp2b* and *laf/alk8* fish suggests that semicircular canal duct outgrowth requires BMP signalling through Alk8. *smad5* codes for an activator Smad, a downstream component of the BMP signal transduction cascade: normal vestibular function in rescued *sbn/smad5* mutants suggests that other activator Smads (Smad1 and Smad8) can compensate for the loss of Smad5 function during semicircular canal morphogenesis.

### Bmp2 has a conserved role in semicircular canal morphogenesis

Details of the early stages of semicircular canal morphogenesis differ between vertebrate species. In mammals and birds, a canal pouch (a flattened outpocketing of the otic vesicle) is formed first. The sides of the pouch then come together to form a fusion plate, and cell death, epithelial resorption or epithelial-to-mesenchymal transition (or a combination of these processes) at the fusion plate results in the formation of the canal [39]–[44]. In zebrafish, there are no pouches; instead, epithelial projections (topologically equivalent to the flattened sides of the canal pouches in amniotes) move towards the centre of the otic vesicle, and meet at a small fusion plate to form a pillar, around which the lumen of the semicircular canal runs [19], [45]. Fusion plates form in the zebrafish between 60 and 72 hpf [19], although a concentration of actin at the fusion plate is still evident at 24 dpf (CM, unpublished observation).

The initial arrangement of semicircular canals in the zebrafish, compared to that in amniotes, is very compact; much further outgrowth is required—to expand both the lumen and circuit radii of each canal, and to form the ampullae—before the canals attain their final shape and become functional [46], [47]. The ears of rescued *swr* fish appear normal during the stages of epithelial projection outgrowth and fusion plate formation (up to 7 dpf). We cannot exclude a role for *bmp2b* during these early stages, as protein from the rescuing RNA may persist during this time. However, our results reveal a definite requirement during the later stages of semicircular canal outgrowth and remodelling. Interestingly, in rescued *laf/alk8* mutants, the heart appears normal during embryonic and larval stages, and both the heart defect and behavioural phenotype appear together at about 2–4 weeks of age (K. Mintzer and MCM, unpublished observations). The zebrafish is known to undergo a larval to juvenile metamorphosis at this stage, involving substantial changes to the morphology of several organ systems, together with alterations to physiology and behaviour [48] (and references within). It is therefore possible that BMP signalling is redeployed in the ear during this metamorphosis to mediate the growth and maturation of the semicircular canal system.

Despite the differences between zebrafish and avian ear morphogenesis, and the fact that the ears of rescued *swr* fish appear normal during embryonic stages, it is interesting that the phenotype of rescued adult *swr* inner ears is similar to that observed when the FGF inhibitor SU5402 was applied to chicken inner ears prior to canal pouch formation, which resulted in the down-regulation of both FGF signalling and *Bmp2* expression [5]. Our data therefore suggest a conserved role for Bmp2 in semicircular canal development across the species from mammals and birds to zebrafish.

## Materials and Methods

### Ethics Statement

All animal work was conducted according to relevant national and international guidelines.

### Zebrafish stocks

Wild-type fish used were the Tübingen strain (Tü); *swr* alleles used were *swr^ta72^*, *swr^tdc24^* and *swr^tc300^*. All adults used were at least 6 months old and of normal size. Embryonic stages are given as hours post-fertilisation (hpf) at 28.5°C and as somite stages for embryos younger than 24 hpf [49], [50].

### mRNA Injections

5-methylguanosine-capped full length sense mouse *smad5* mRNA or *bmp2b* mRNA was injected into 1–2 cell embryos from a *swr^+/−^*×*swr^+/−^* mating as previously described [9], [11], [49], [51]. Alternatively, for *swr^tdc24^*, the progeny of two adult rescued *swr^−/−^* homozygotes were injected [52]. *pCS2+-smad5* template was linearised with *Acc*651, *p64T-bmp2b* was linearised with *Xba*1 and RNA for both was transcribed using SP6 polymerase according to standard protocols [53]. For *bmp2b* rescue, 94% of injected mutant embryos are rescued to a wild-type or weakly dorsalised phenotype (∼50%) or a ventralised phenotype (∼50%) [11]. For *smad5* rescue, efficiency is similar to that already described for *smad1* [11]: about 80% of injected mutant embryos are rescued to a wild-type or a weakly dorsalised (viable) phenotype. Only those animals rescued to a wild-type phenotype were analysed here. Injected embryos were processed for phalloidin staining (see below) or raised to adulthood and rescued *swr^−/−^* individuals identified by PCR genotyping as previously described (*swr^ta72^*: [37]; *swr^tc300^*: [54]). DNA was obtained from fin clips according to standard procedures [49].

### Dorsal light reflex

In fish, the tendency to orient dorsal side up relies primarily on vestibular inputs, but visual inputs can compensate for a loss of vestibular function [55]. When a fish tank is illuminated from the side in a darkened room, fish that cannot detect gravitoinertial stimuli will orient their dorsal side towards the light source (the dorsal light reflex), while there is no immediate effect on wild-type fish. The dorsal light reflex of five adult *bmp2b*-rescued *swr^ta72^* fish was assessed as described [17]. The test was repeated three times for each fish.

### Histological analysis

Histology was carried out on *swr^ta72^* fish rescued with *bmp2b* RNA and *swr^tdc24^* and *swr^tc300^* rescued with *smad5* RNA. Heads of adult fish were fixed for 1 to 2 days at 4° C in 4% paraformaldehyde, treated with 0.12 M EDTA for three days to remove otoliths, dehydrated through an ethanol series, and embedded in JB4 resin (Polysciences), before sectioning at 10 µm using a steel knife. Sections were stained with toluidine blue and mounted in DePeX (Sigma) before photography using a Camedia (C-3030ZOOM) camera, AnalySIS software and a BX51 compound microscope (Olympus). Images were assembled and painted using Adobe Photoshop and reconstructions produced using Amira 4.0 software (Visage Imaging).

### FITC-Phalloidin staining

Embryos were fixed overnight in 4% paraformaldehyde, rinsed in PBS, and whole-mount stained with FITC-conjugated phalloidin as described previously [19], mounted in Vectashield (Vector laboratories) and imaged using a Leica SP confocal microscope. Ears were dissected for dorsal views.

## Supporting Information

Video S1 360° rotation of the reconstructed adult swr/bmp2b mutant ear shown in Fig. 2B
(2.70 MB MOV)

Video S2 360° rotation of the reconstructed adult wild-type ear shown in Fig. 2C
(2.78 MB MOV)
